# RNA and DNA Targeting by a Reconstituted *Thermus thermophilus* Type III-A CRISPR-Cas System

**DOI:** 10.1371/journal.pone.0170552

**Published:** 2017-01-23

**Authors:** Tina Y. Liu, Anthony T. Iavarone, Jennifer A. Doudna

**Affiliations:** 1 Department of Molecular and Cell Biology, University of California, Berkeley, California, United States of America; 2 Howard Hughes Medical Institute, University of California, Berkeley, California, United States of America; 3 Department of Chemistry, University of California, Berkeley, California, United States of America; 4 Innovative Genomics Initiative, University of California, Berkeley, California, United States of America; 5 MBIB Division, Lawrence Berkeley National Laboratory, Berkeley, California, United States of America; 6 California Institute for Quantitative Biosciences, University of California, Berkeley, California, United States of America; Saint Louis University, UNITED STATES

## Abstract

CRISPR-Cas (clustered regularly interspaced short palindromic repeats-CRISPR-associated) systems are RNA-guided adaptive immunity pathways used by bacteria and archaea to defend against phages and plasmids. Type III-A systems use a multisubunit interference complex called Csm, containing Cas proteins and a CRISPR RNA (crRNA) to target cognate nucleic acids. The Csm complex is intriguing in that it mediates RNA-guided targeting of both RNA and transcriptionally active DNA, but the mechanism is not well understood. Here, we overexpressed the five components of the *Thermus thermophilus* (*T*. *thermophilus*) Type III-A Csm complex (TthCsm) with a defined crRNA sequence, and purified intact TthCsm complexes from *E*. *coli* cells. The complexes were thermophilic, targeting complementary ssRNA more efficiently at 65°C than at 37°C. Sequence-independent, endonucleolytic cleavage of single-stranded DNA (ssDNA) by TthCsm was triggered by recognition of a complementary ssRNA, and required a lack of complementarity between the first 8 nucleotides (5′ tag) of the crRNA and the 3′ flanking region of the ssRNA. Mutation of the histidine-aspartate (HD) nuclease domain of the TthCsm subunit, Cas10/Csm1, abolished DNA cleavage. Activation of DNA cleavage was dependent on RNA binding but not cleavage. This leads to a model in which binding of an ssRNA target to the Csm complex would stimulate cleavage of exposed ssDNA in the cell, such as could occur when the RNA polymerase unwinds double-stranded DNA (dsDNA) during transcription. Our findings establish an amenable, thermostable system for more in-depth investigation of the targeting mechanism using structural biology methods, such as cryo-electron microscopy and x-ray crystallography.

## Introduction

CRISPR (clustered regularly interspaced short palindromic repeats) loci and Cas (CRISPR-associated) genes constitute an RNA-guided adaptive immune system used by bacteria and archaea to protect against phage and plasmid infection [1–4]. They are present in ~50% of bacteria and most archaea [4]. In general, short fragments of an invader’s genome are integrated into the host genome at a CRISPR locus, transcribed into pre-CRISPR RNAs (pre-crRNAs), and processed into individual, mature crRNA molecules that assemble with multiple Cas proteins (Class 1 systems) or a single Cas protein (Class 2 systems) into effector complexes [4]. These complexes then mediate degradation of foreign DNA or RNA that have sequences bearing complementarity to the crRNA.

**Fig 1:**
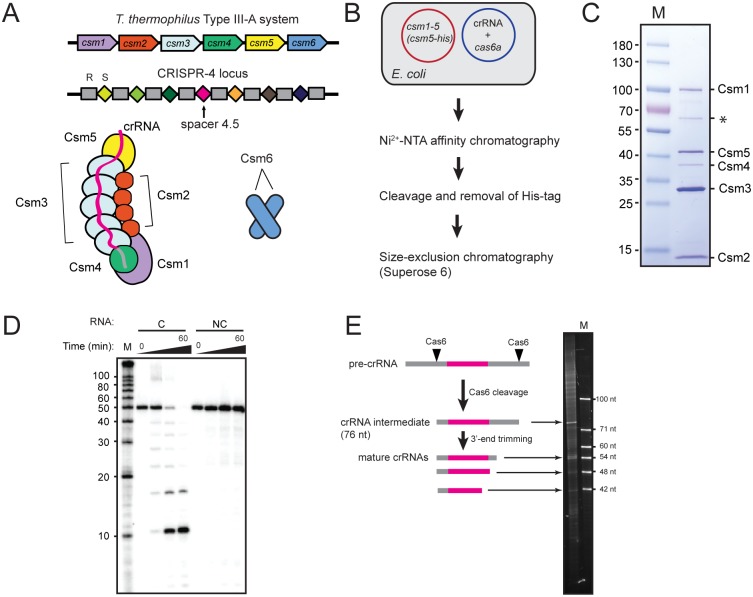
Reconstitution and RNA cleavage activity of a *T*. *thermophilus* Csm complex (TthCsm) purified from *E*. *coli* with a defined crRNA species. **(A)** Components of the CRISPR locus and effector complexes of the *T*. *thermophilus* Type III-A Csm system. The complex is shown with 5 copies of Csm3 and 4 copies of Csm2, but complexes with different numbers of these two subunits also exist. The CRISPR-4 locus associated with the system is shown (repeat is designated by R and spacer by S). The spacer 4.5 used for complex reconstitution encodes for one of the most abundant crRNAs found in the host organism [21]. (**B**) Reconstitution and purification of TthCsm in *E*. *coli*. A plasmid containing genes encoding for Cas10/Csm1, and Csm2-5, with a His_10_ tag on Csm5, was co-transformed into *E*. *coli* with a plasmid containing genes for expression of *T*. *thermophilus* Cas6A and a single CRISPR array containing one copy of spacer 4.5. The purification steps are indicated. (**C**) TthCsm was subjected to SDS polyacrylamide gel electrophoresis (SDS-PAGE) analysis following purification. Csm subunits are labeled, and a molecular weight ladder (M) is in the left lane (masses are given in kilodaltons). A GroEL contaminant (asterisk) was also identified by mass spectrometry (S2 Table). (**D**) TthCsm-mediated cleavage of a complementary (C) or noncomplementary (NC) ^32^P-labeled ssRNA oligonucleotide was tested in the presence of 2 mM MgCl_2_. Samples taken at 0, 5, 30, and 60 minutes after TthCsm addition were analyzed by denaturing PAGE. (**E**) Schematic of crRNA processing in Type III CRISPR-Cas systems is shown on the left. Pre-crRNAs are cleaved by Cas6 to generate an intermediate, which is then trimmed at the 3’-end, resulting in mature crRNAs. On the right, nucleic acids associated with the Csm complex were extracted and analyzed by denaturing PAGE. An ssDNA oligonucleotide ladder (M) was loaded in the right-most lane and nucleotide lengths are indicated.

Type III CRISPR-Cas is a widespread Class 1 system, comprising about a quarter of all CRISPR systems in bacteria and a third in archaea [1]. They use multisubunit effector complexes composed of several different Cas proteins bound to a crRNA molecule to recognize and target nucleic acids [5]. Effector complexes from the two major Type III subtypes, III-A and III-B, are called Csm and Cmr, respectively. In these complexes, Cas10, also known as Csm1 or Cmr2, and Csm4/Cmr3 are positioned at the foot of the complex, while two filaments, composed of Csm3/Cmr4 and Csm2/Cmr5 subunits, wind around each other to form the backbone and belly [5]. A single protein in Type III-A systems, Csm5, or two proteins in Type III-B systems, Cmr1 and Cmr6, cap the backbone filament at the head of the complex [5] (Fig 1A). The Cas10 protein typically contains an HD nuclease domain and palm polymerase domain, and is thought to be the subunit responsible for DNA cleavage activity [1,6–11]. The overall architecture of Type III complexes is similar to that of the more well-studied Type I “Cascade” complex, but unlike Cascade, which recruits a trans-acting Cas3 nuclease/helicase to degrade double-stranded DNA (dsDNA), the catalytic components are constitutively present in Type III complexes [6,10–16]. In this respect, Type III systems are more akin to the single-component, multi-domain Class 2 effector proteins like Type II Cas9, Type V Cpf1, and Type VI C2c2, which have intrinsic nuclease activities [17–20].

The nucleic acid targeting mechanism of Type III systems has long been a subject of controversy, since it was first reported that a Type III-A system targeted DNA *in vivo* while a Type III-B system targeted ssRNA *in vitro* [14,22]. Subsequent studies showed that several Type III-A systems also target ssRNA, and the *Staphylococcus epidermidis (S*. *epidermidis)* Csm was also shown to target transcriptionally active regions of DNA both *in vivo* and *in vitro* [6,21,23,24]. This led to the hypothesis that the Csm complex might recognize unwound ssDNA at the site of transcription [6,21,24]. Recent studies of several Type III-A and III-B systems have demonstrated that RNA binding activates cleavage of ssDNA, leading to a proposed model in which a growing messenger RNA would tether the Type III effector complex to the site of transcription and trigger degradation of the DNA unwound by the RNA polymerase [9–11]. However, whether this is indeed the case is not known, as no direct interaction between a Type III complex and the transcription bubble (the site where the RNA polymerase has unwound the DNA) has been reported. In addition, the molecular basis of DNA and RNA recognition during transcription by the Type III-A complex is poorly understood. Studies suggest that the catalytic residues for RNA cleavage are in the backbone subunit, Csm3, and those for DNA cleavage are in either the HD or Palm polymerase domain of the Cas10/Csm1 subunit [5,6,25]. The similarity between the architecture of Cmr and Csm complexes suggests that Csm may bind its RNA target in a manner similar to that revealed by structures of the Cmr complex [10,26,27]. However, this remains a hypothesis that must be tested by empirical studies. It is also not clear how the Csm complex would access and bind unwound ssDNA at the transcription bubble. Thus, further studies are required to elucidate the true target of Type III complexes.

The Type III-A Csm complex of the bacterium *T*. *thermophilus* (TthCsm) presents an attractive system for the study of transcription-dependent DNA and RNA targeting, as it is thermostable and the structure of the elongating RNA polymerase from this bacterium has previously been determined using X-ray crystallography [28]. In addition, the TthCsm complex is amenable to negative stain EM analysis, making it an attractive candidate for structural studies using cryo-EM [21]. However, DNA targeting has not yet been demonstrated for the TthCsm complex, and endogenous TthCsm complexes are associated with many different crRNA guide sequences, which may hinder attempts to isolate complexes bound to a single RNA and DNA target sequence [21]. Here, we reconstituted a thermophilic Csm complex from *T*. *thermophilus* by co-overexpression of the Csm subunit proteins with a single crRNA sequence and the crRNA processing factor, Cas6, in *E*. *coli*, and purified it using an affinity tag on one of the subunits. Using this system, we demonstrate that the reconstituted *T*. *thermophilus* Csm complex (TthCsm) contains processed and mature crRNAs, and is able to bind and cleave complementary RNA at high temperatures. Our results also show that this Type III-A complex is capable of robust ssDNA cleavage when provided with a complementary ssRNA target, thus explaining why DNA cleavage was not observed in previous studies of *T*. *thermophilus* Csm complexes [21,29]. DNA cleavage by the TthCsm also requires noncomplementarity between the first 8 nucleotides (nt) of the crRNA (5’ tag) and the 3’ flanking region of the target RNA. We also found that the substrate requirements and catalytic site of DNA cleavage of this complex are similar to that of several Type III-A and III-B systems from other species [10,11,15]. Thus, use of this reconstituted system for structural analysis would provide universal insights into target recognition and cleavage by Type III systems.

## Materials and Methods

### Cloning and plasmids

To construct a vector expressing the complex components (pCDF-TtCsm), codon-optimized *T*. *thermophilus cas10/csm1* (TTHB147), *csm2* (TTHB148), *csm3* (TTHB149), *csm4* (TTHB150), and His-tagged *csm5* (TTHB151) were ordered from GeneArt^®^ (Thermofisher Scientific) either as Strings^®^ gene fragments or cloned genes and subcloned into the pCDF-1b vector using overlap polymerase chain reaction (PCR) and Gibson assembly. An additional T7 promoter, *lac* operator, and ribosome-binding site (rbs) was placed before *csm3*, and rbs sequences were placed before *csm2*, *csm4*, and *csm5*. The *csm5* gene sequence was followed by a sequence encoding for a human rhinovirus (HRV) 3C protease cleavage site (SAVELFQGP) and a decahistidine tag (His_10_-tag). To construct pACYC-TtCas6A-crRNA4.5, a repeat-spacer-repeat array containing the 5^th^ spacer from the CRISPR4 locus of *T*. *thermophilus* HB8 and the codon-optimized *cas6A* (TTHA0078) from *T*. *thermophilus* HB8 were cloned after T7 promoters of the pACYCDuet vector to generate pACYC-TtCas6A-crRNA4.5. Mutagenesis of individual subunits was performed using the Q5 site-directed mutagenesis kit (New England Biolabs). Sequences of all constructs were verified by DNA sequencing (UC Berkeley Sanger Sequencing Facility).

### Protein expression and purification

For expression of TthCsm, the pCDF-TtCsm and pACYC-TtCas6-crRNA4.5 were both transformed into the BL21(λDE3) strain of *Escherichia coli* (*E*. *coli)*. Cells were grown in the presence of streptomycin and chloramphenicol in LB (Luria-Bertani) medium to an optical density at 600 nm of 0.6 and expression was induced by addition of 0.5 mM IPTG (isopropyl β-D-1-galactopyranoside). After overnight growth at 16°C, the cells were harvested and resuspended in lysis buffer (25 mM HEPES, pH 7.5, 150 mM KCl, 5% (v/v) glycerol, 10 mM imidazole, 1 mM TCEP (Tris(2-carboxylethyl)phosphine), 0.01% (v/v) Triton X-100) supplemented with 1 mM PMSF (phenylmethylsulfonyl fluoride) and an EDTA (ethylenediaminetetraacetic acid)-free protease inhibitor cocktail tablet (Roche). Cells were lysed by sonication and the cell lysate was clarified by centrifugation. TthCsm was isolated from the lysate using Ni^2+^-NTA superflow resin (Qiagen, Inc.), washed sequentially with Wash Buffer 1 (25 mM HEPES, pH 7.5, 500 mM KCl, 5% glycerol, 20 mM imidazole, 1 mM TCEP, 2 mM ATP, 10 mM MgCl_2_) and Wash buffer 2 (same as Wash buffer 1, but without ATP or MgCl_2_), and eluted with 25 mM HEPES, pH 7.5, 150 mM KCl, 5% glycerol, 300 mM imidazole, 1 mM TCEP). The eluted protein was dialyzed against 25 mM HEPES, pH 7.5, 150 mM NaCl, 5% glycerol, 1 mM TCEP and the His-tag was cleaved from Csm5 with the HRV 3C protease. The cleaved His-tag and other impurities were removed using Ni^2+^-NTA resin. The complex was further purified by size-exclusion chromatography using a Superose 6 column (GE Healthcare); fractions containing the subunits at the highest concentration were collected and concentrated.

### DNA and RNA substrates

Synthetic oligonucleotides were ordered from Integrated DNA Technologies, purified by denaturing polyacrylamide gel electrophoresis (PAGE, 7 M Urea). All oligonucleotides were 5′ labeled with [γ-^32^P]-ATP using T4 polynucleotide kinase for cleavage and binding assays. Sequences of DNA and RNA substrates used are given in S1 Table.

### RNA cleavage assays

We performed RNA cleavage assays with TthCsm and a ^32^P-radiolabeled ssRNA oligonucleotide mixed to a final concentration of 100 nM and 20 nM, respectively, in 25 mM HEPES, pH 7.5, 150 mM NaCl, 10 mM DTT (dithiothreitol) and 2 mM MgCl_2_. For some experiments, 200 nM TthCsm and 5 nM ^32^P-radiolabeled ssRNA oligonucleotide were mixed in a buffer of 25 mM Tris, pH 7.5, 40 mM KCl, 1 mM TCEP, 1 mM EDTA instead. The reactions were warmed to 65°C for 10 min. Reactions were then initiated by addition of 5 mM MnCl_2_. Samples were taken out at the time points indicated, quenched with Gel Loading Buffer II (ThermoFisher Scientific), and heated at 95°C for 5 min. Cleavage products were resolved on a 15% denaturing polyacrylamide gel, and visualized by phosphorimaging. An ssRNA Decade^™^ Marker (ThermoFisher Scientific) was loaded where indicated for size estimation.

### Liquid chromatography-tandem mass spectrometry

Trypsin-digested protein samples were analyzed by liquid chromatography-tandem mass spectrometry (LC-MS/MS) using a Thermo-Dionex UltiMate3000 RSLCnano liquid chromatograph that was connected in line with an LTQ-Orbitrap-XL mass spectrometer equipped with a nanoelectrospray ionization source (Thermo Fisher Scientific, Waltham, MA). Data acquisition was controlled using Xcalibur software (version 2.0.7, Thermo) and data analysis was performed using Proteome Discoverer software (version 1.3, Thermo). LC-MS/MS method details have been published elsewhere [30].

### Native nanoelectrospray ionization mass spectrometry

Approximately 5 μM TtCsm was subjected to five rounds of buffer exchange into 1 M ammonium acetate, with 5% (v/v) glycerol, using 10,000 MWCO (molecular weight cut-off) Corning Spin-X concentrators. Native nanoelectrospray ionization mass spectrometry (nanoESI-MS) measurements were obtained in the positive ion mode on a Synapt G2-S*i* mass spectrometer equipped with a nanoESI source (Waters, Milford, MA). Mass spectrometry data acquisition and processing were performed using MassLynx software (version 4.1, Waters).

### Extraction and analysis of crRNA from the complex

The purified complex was subjected to phenol-chloroform extraction, followed by ethanol precipitation. The extracted nucleic acid was analyzed by 10% denaturing PAGE (7 M Urea) and visualized by SYBR Gold staining. An ssDNA ladder containing synthetic oligonucleotides of length 100, 71, 60, 54, 48, and 42 nt from IDT was used as a marker.

### Electrophoretic mobility shift assays (EMSA)

Varying concentrations of TthCsm were incubated with 0.5 nM ^32^P-radiolabeled ssRNA at 65°C for 20 min. The binding buffer contained 25 mM HEPES, pH 7.5, 150 mM NaCl, 5% glycerol, 1 mM TCEP, 1 mM EDTA. For competition assays, increasing concentrations of unlabeled oligonucleotides of 0, 5, 50, 100, 500, 1000 nM were mixed with 0.5 nM ^32^P-radiolabeled ssRNA and then combined with 100 nM Csm at the conditions described above. For EMSA experiments, concentrations of TthCsm ranging from either 0–300 nM (0, 0.1, 0.3, 1, 3, 10, 30, 100, 300 nM) were used, or 0–1200 nM (0, 0.1, 0.3, 1, 3, 10, 30, 100, 300, 600, 900, 1200 nM) were used, where indicated. Binding reactions were analyzed by 6% native PAGE at 4°C, and the ^32^P-radiolabeled oligonucleotide was visualized by phosphorimaging. EMSA experiments on mutants or RNA variants tested in DNA cleavage assays were done as described above, but with a buffer more similar to that used for DNA cleavage (25 mM Tris, pH 7.9, 40 mM KCl, 5% glycerol, 1 mM TCEP, 1 mM EDTA).

### DNA cleavage assays

For DNA cleavage assays, 200 nM Csm, 200 nM ssRNA, and 5 nM ^32^P-radiolabeled DNA were mixed in 25 mM Tris, pH 7.9, 40 mM KCl, 1 mM TCEP, 1 mM EDTA, and warmed for 65°C for 10 minutes. Reactions were then initiated by addition of 5 mM MnCl_2_. Samples were taken out at the time points indicated, quenched with Gel Loading Buffer II (ThermoFisher Scientific), and heated at 95°C for 3 min. Cleavage products were resolved by 10% denaturing PAGE (7 M Urea), and visualized by phosphorimaging. A ssRNA Decade^™^ Marker (ThermoFisher Scientific) was loaded where indicated for size estimation of the cleavage products.

## Results

### Reconstitution of a thermophilic Type III-A Csm complex

The *Thermus thermophilus* HB8 strain harbors three CRISPR systems: III-A, III-B, and I-E [21]. The six *cas* genes of the Type III-A system (*csm1-6*) encode five components of the effector complex, and a separate nonspecific RNase (Csm6) [21,31] (Fig 1A). The crRNA sequences associated with the endogenous TthCsm all contain the same sequence of 8 nucleotides at the 5′ end (5′ tag), but are derived from 7 different CRISPR loci in the genome [21]. Processing of crRNAs likely involves an initial cleavage event by a Cas6 ribonuclease that is encoded elsewhere in the genome, followed by trimming of the 3′ end of the crRNA to variable lengths [32,33]. There are three genes that encode for Cas6 proteins in the *T*. *thermophilus* genome, but only Cas6A and Cas6B process crRNAs associated with *T*. *thermophilus* Type III-A Csm complexes [21,34]. Thus, to reconstitute TthCsm with a single, defined crRNA sequence in an organism that is genetically manipulable and can be easily grown under standard laboratory conditions, we cloned codon-optimized *csm1-5*, *cas6A*, and a single repeat-spacer-repeat array from the CRISPR-4 locus in *T*. *thermophilus* into expression vectors, and introduced them for expression in *E*. *coli* (Fig 1B). We isolated the reconstituted TthCsm using a His_10_ affinity tag on Csm5 and removed large aggregates by size-exclusion chromatography (Fig 1B). To confirm the presence and purity of all five Csm subunits, we performed SDS-PAGE analysis and did in-solution trypsin digestion and nanoscale liquid chromatography-tandem mass spectrometry (LC-MS/MS) analysis on the sample (Fig 1C and S2 Table). Peptides from all five subunits were detected, as well as a contaminant, the chaperonin, GroEL, which corresponds to the ~60 kDa band detected by SDS-PAGE and Coomassie Blue staining (Fig 1C and S2 Table). To test whether the complex could cleave ssRNA complementary to the expressed crRNA sequence, we tested whether the purified sample could cleave an ssRNA substrate complementary to the crRNA. The reconstituted TthCsm catalyzed cleavage of the complementary ssRNA at every 6 nucleotides, as observed for other Csm complexes (Fig 1D) [6,21,23]. A non-complementary RNA sequence was not cleaved (Fig 1D), indicating that we had assembled a functional TthCsm in a heterologous *E*. *coli* system.

Next, we sought to determine the lengths of the crRNAs associated with the reconstituted complex. Purified samples of endogenous TthCsm complexes were reported to associate with crRNAs ranging from ~35–53 nt long, with a trend toward lengths that are spaced by 5–6 nucleotides [21]. To determine the nucleic acid content of the reconstituted TthCsm, we extracted RNA from the sample and analyzed it by denaturing gel electrophoresis, revealing a species slightly larger than the 71 nt-long marker that likely corresponds to the 76-nt Cas6-processed crRNA transcript (Fig 1E). Shorter crRNAs of approximately 54, 48, and 42 nucleotides in length were also observed (Fig 1E). This result indicates that a crRNA expressed from a single spacer sequence from *T*. *thermophilus* can be processed by Cas6 and trimmed to their mature sizes in *E*. *coli*. It also indicates that a crRNA expressed from a single spacer sequence is processed to several different lengths.

To determine the molecular composition of the reconstituted TthCsm, we used native nanoelectrospray ionization mass spectrometry (nanoESI-MS) to determine its mass (S1 Fig). Four complexes were detected, and based on the homologous architecture of Csm complexes to structures of the Cmr complex, it is likely that the complex contains single copies of Cas10/Csm1, Csm4, and Csm5, and variable numbers of the Csm3 and Csm2 subunits (S1 Fig) [13,21,26,27,35]. The 390.4 kDa complex contains single copies of Cas10/Csm1, Csm4 and Csm5, six Csm3 subunits, four Csm2 subunits and a 54 nt-long crRNA. The 338.5 kDa complex is similar but contains four Csm3 subunits and three Csm2 subunits and a 40–41 nucleotide crRNA (S1 Fig). The 299.5 kDa and 248.3 kDa complexes are similar to the 390.4 and 338.5 complexes but lack Cas10/Csm1 in each case. This suggests that the Cas10/Csm1 subunit is more weakly associated with the complex than the other subunits.

**Fig 2:**
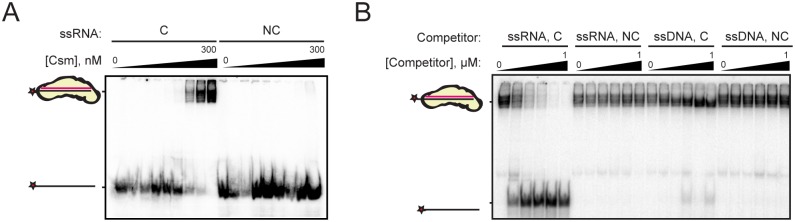
TthCsm specifically recognizes and binds ssRNA through complementarity to its crRNA. (**A**) An increasing concentration of TthCsm, from 0–300 nM, was incubated with 0.5 nM ^32^P-labeled ssRNA target that was complementary (C) or noncomplementary (NC) to the crRNA guide sequence, and analyzed by an EMSA. (**B**) 100 nM TthCsm was incubated with 0.5 nM ^32^P-labeled target ssRNA and increasing concentrations of unlabeled complementary ssRNA (ssRNA, C), noncomplementary ssRNA (ssRNA, NC), complementary ssDNA (ssDNA, C), or noncomplementary ssDNA (ssDNA, NC) competitor (0–1 μM). Samples were assayed for binding of the probe using an EMSA, as in (A).

To test the specificity of RNA binding by TthCsm to its target RNA, we performed electrophoretic mobility shift assays (EMSAs) with ^32^P-radiolabeled ssRNA substrates containing a 40-nt sequence complementary or noncomplementary to the crRNA and random 5-nt-long flanking regions (Fig 2A). EDTA was included in the binding reaction to prevent cleavage of the RNA by the complex. A mobility shift of the complementary, but not non-complementary, RNA occurred when increasing concentrations of TthCsm were added, indicating that complex binding to RNA is specific. An unlabeled complementary ssRNA also competed efficiently with the labeled ssRNA for binding to TthCsm, while a noncomplementary substrate did not (Fig 2B). However, neither a complementary nor a random ssDNA oligonucleotide was able to compete with the complementary ssRNA for binding to TthCsm, suggesting that the complex only recognizes RNA through base-pairing interactions with its crRNA (Fig 2B).

**Fig 3:**
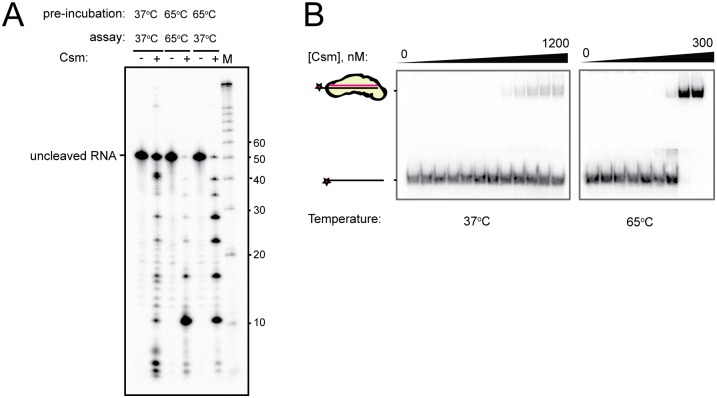
TthCsm binds and cleaves its ssRNA target optimally at high temperatures. **(A)** TthCsm was pre-incubated in the absence of metal ions with complementary ssRNA at either 65°C or 37°C (pre-incubation), and then cleavage was initiated by addition of MgCl_2_ and continued at either 65°C or 37°C, as indicated (assay). Reactions with (+) or without (-) TthCsm added are shown. **(B)** TthCsm was incubated with a ^32^P-labeled complementary ssRNA target at 65°C or 37°C, and binding was measured by an EMSA, as in Fig 2A. TthCsm was added to a concentration of 0–1200 nM at 37°C or 0–300 nM at 65°C.

Since *T*. *thermophilus* is a thermophilic bacterium and grows optimally at temperatures of ~65–75°C, we investigated how temperature affects RNA cleavage and binding by TthCsm. RNA cleavage was minimal at 37°C, but robust at 65°C (Fig 3A); target RNA pre-incubation with TthCsm at 65°C in the absence of metal ions, followed by cooling to 37°C before addition of Mg^2+^, resulted in a somewhat higher level of RNA cleavage than the reaction performed entirely at 37°C. To determine if this was due to impaired RNA binding at lower temperatures, we compared the binding affinity of TthCsm for a ^32^P-radiolabeled, complementary ssRNA target at 37°C and 65°C using an EMSA (Fig 3B). The amount of RNA that was gel-shifted at 37°C was significantly less than at 65°C (Fig 3B). This shows that both RNA binding and cleavage by TthCsm are more efficient at high temperatures. The complex may be unable to undergo conformational changes necessary for target binding at lower temperatures, or the GC-rich target RNA may form stable secondary structures at the lower temperature that TthCsm cannot unwind. Taken together, these data show that the reconstituted TthCsm specifically recognizes ssRNA, but not ssDNA, through complementarity with the crRNA.

### TthCsm-catalyzed ssDNA cleavage requires crRNA and a complementary ssRNA

**Fig 4:**
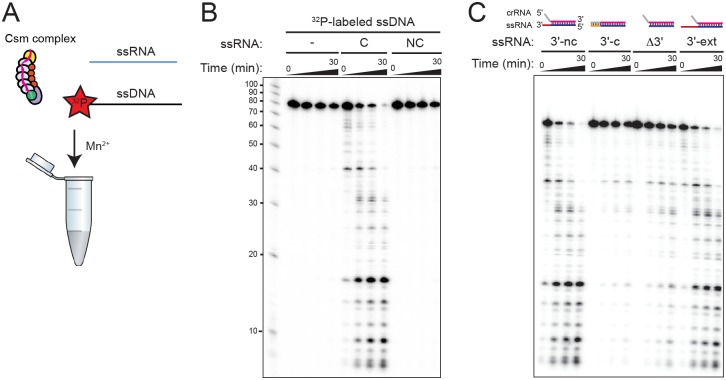
RNA-guided ssDNA cleavage by TthCsm. **(A)** Schematic of the DNA cleavage reaction. TthCsm was mixed with 5′-^32^P-labeled ssDNA oligonucleotide and an unlabeled complementary ssRNA oligonucleotide in the presence of 1 mM EDTA. The reaction was initiated by the addition of 5 mM MnCl_2_. **(B**) TthCsm-mediated ssDNA cleavage was tested in the presence of complementary (C) or noncomplementary (NC) ssRNA, as described in (A). Time points were taken at 0, 10, 15, and 30 min, and cleavage products were analyzed by denaturing PAGE. **(C)** As in (B), but using unlabeled ssRNA substrates that are complementary to the crRNA guide sequence, but have an 8 nt-long 3′ flanking region that is either noncomplementary (3′-nc), complementary (3′-c), truncated (Δ3′), or both extended to 20 nt long and noncomplementary (3′-ext).

The endogenous TthCsm was reported to cleave complementary ssRNA, but not ssDNA or dsDNA oligonucleotides [21]. Using the reconstituted TthCsm, we investigated whether DNA cleavage might occur under different conditions than those tested previously [21]. Since DNA cleavage by the mesophilic *S*. *epidermidis* Csm complex requires active transcription across the target, and binding of complementary RNA has been shown to be required for ssDNA cleavage by Type III-A and III-B complexes from other species, we wondered if targeting by TthCsm might require both DNA and RNA to be present [6,10,11,15]. TthCsm did not cleave ^32^P-radiolabeled ssDNA alone in the presence of Mg^2+^ (S2 Fig). However, addition of ssRNA complementary to the crRNA, but not a non-complementary ssRNA, triggered slow DNA cleavage (S2 Fig). We found that DNA cleavage was more robust in the presence of MnCl_2_, compared to in MgCl_2_, but was still dependent on the presence of a complementary ssRNA sequence (Fig 4A and 4B). In the absence of Mn^2+^, some DNA cleavage was observed when the complementary RNA was present (0 min time point, Fig 4B), possibly due to metal ions that remain associated with TthCsm during purification. This indicates that TthCsm catalyzes cleavage of ssDNA when provided with a complementary “activator” ssRNA.

Self versus non-self discrimination in Type III-A systems was proposed to rely on detection of complementarity between a DNA target’s 3′ flanking sequence and the 5′ tag of the crRNA associated with the *S*. *epidermidis* Csm; complementarity to the repeat-derived 5′ tag of the crRNA would prevent cleavage at the host’s CRISPR loci [36]. A recent study of a Csm complex from *Streptococcus thermophilus* (S. thermophilus) showed that the complementarity is actually detected in the 3′ flanking sequence of the RNA target, rather than in the DNA [10]. To test whether this was the case for the TthCsm, we tested ssRNA substrates that contained the 40-nt complementary target, but different 3′ flanking sequences. Substrates that had an 8 nt-long, noncomplementary 3′ flanking region activated the TthCsm for DNA cleavage (Fig 4C). When the 3′ flanking region was completely complementary to the 5′ tag (3′-c), the DNA cleavage activity of TthCsm was inhibited, suggesting that recognition of self DNA occurs through recognition of the 3′ flanking region of the RNA (Fig 4C). Deletion of the 3′ flanking region of the RNA also inhibited DNA cleavage (Δ3′), while extension of a noncomplementary 3′ flanking region to 20 nt in length allowed DNA targeting by TthCsm (3′-ext) (Fig 4C). This suggests that the 3′ flanking region of the RNA may interact with the complex to activate its DNA cleavage activity; disruption of this interaction can occur by base-pairing with the 5′ tag or truncation may prevent this interaction. To test whether the 3′ flanking sequence functions independently from the rest of the RNA, we added an 8 nt-long ssRNA oligonucleotide that was not complementary to the crRNA 5′ tag of TthCsm in combination with the truncated (Δ3′) RNA and tested whether the complex could cleave DNA (S3 Fig**)**. We did not observe a stimulatory effect of the 8-nt ssRNA on DNA cleavage, even at 100-fold molar excess over the truncated ssRNA and TthCsm (S3 Fig). In fact, the oligo appeared to slightly diminish the efficiency of DNA cleavage with the truncated RNA, suggesting that the oligo may not be binding to the same place as the attached 3′ flanking sequence. Thus, the physical connection of the 3′ sequence to the rest of the RNA is crucial for activation of DNA targeting, most likely to position it correctly for activation of Cas10/Csm1.

### The Cas10/Csm1 HD domain catalyzes sequence-independent, endonucleolytic DNA cleavage by TthCsm

**Fig 5:**
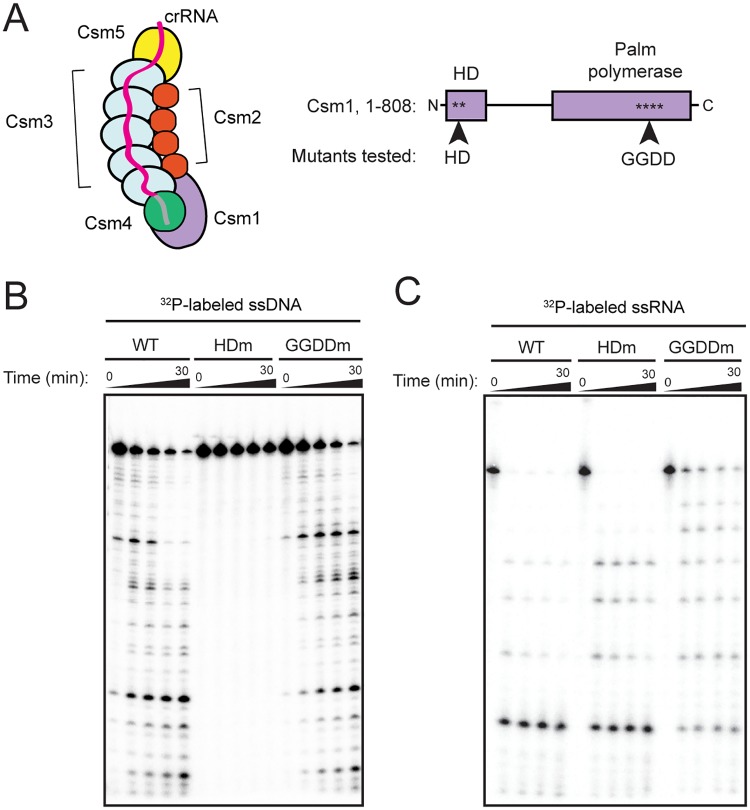
The HD domain is required for ssDNA cleavage by TthCsm. **(A)** Schematic of TthCsm, and domain architecture of the *T*. *thermophilus* Csm1 component protein are shown. The HD and palm polymerase domain are indicated, and the approximate positions of the HD and GGDD mutations tested in (B) and (C) are shown. N and C termini of Csm1 are indicated. (**B**) ssDNA cleavage mediated by the wild-type TthCsm (WT), TthCsm containing an HD domain mutation (H18A/D19A, indicated by HDm), and TthCsm complex containing a palm polymerase domain mutation (G630A/G631A/D632A/D633A, indicated by GGDDm) was tested as in Fig 4B. Time points were taken at 0, 5, 10, 15, and 30 min and analyzed by denaturing PAGE. (**C**) WT, HDm or GGDDm TthCsm complexes were tested for ssRNA cleavage, using same conditions as in (B), but with only complementary, radiolabeled ssRNA added.

To determine the location of the catalytic site of DNA cleavage in TthCsm, we made mutations in two domains of Cas10/Csm1 that could have enzymatic activity, the HD nuclease domain and the palm polymerase domain [5] (Fig 5A). We expressed and purified TthCsm mutant complexes from *E*. *coli* and tested their ability to cleave ssDNA. We found that mutation of the HD motif in Cas10/Csm1 to alanines completely abolished RNA-activated DNA cleavage (Fig 5B). Mutation of the catalytic motif, GGDD, of the palm polymerase domain did not lower the DNA cleavage activity of TthCsm (Fig 5B). We also tested whether these mutations might indirectly affect DNA cleavage by preventing recognition of the ssRNA target. However, both complexes containing these mutant proteins were capable of ssRNA cleavage, indicating that this was not due to impaired activation of DNA cleavage by the ssRNA (Fig 5C). Thus, the HD nuclease domain in the Cas10/Csm1 subunit is responsible for catalyzing ssDNA cleavage.

**Fig 6:**
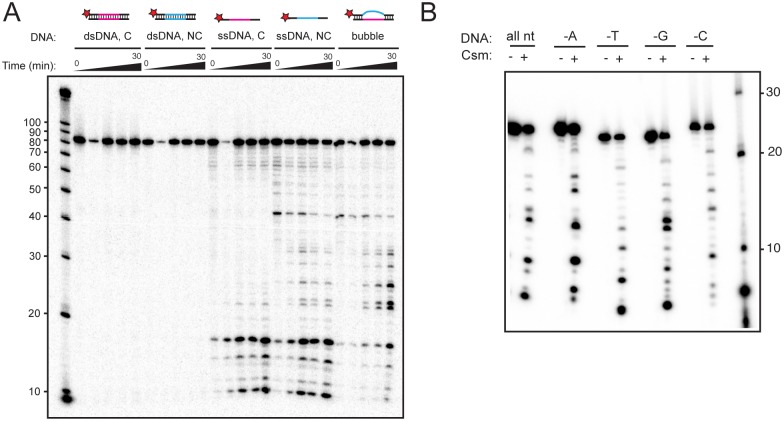
TthCsm-mediated ssDNA cleavage is sequence-independent and endonucleolytic. **(A)** TthCsm-mediated cleavage of a 5′-^32^P-radiolabeled complementary or noncomplementary dsDNA (dsDNA, C or NC), ssDNA (C or NC), or a duplex with a 40-nt long mismatch in the center (bubble DNA) was tested in the presence of complementary target ssRNA, as in Fig 5B. **(B)** TthCsm was incubated with 5′-^32^P-radiolabeled 25 nt-long ssDNA substrates with different sequences in the presence of a complementary target ssRNA and MnCl_2_. Sequences included all four nucleotides (all nt), all except adenines (-A), thymines (-T), guanines (-G), or cytosines (-C). The reaction was carried out for 60 minutes, either with (+) or without (-) TthCsm added.

Next, we wondered whether TthCsm mediates cleavage of ssDNA or dsDNA, and if so, whether it has endonucleolytic activity. Thus, we annealed complementary 88 nt ssDNA oligonucleotides with a melting temperature (T_m_) greater than 65°C, the temperature of the assays, and tested whether TthCsm could cleave these substrates when provided with an activating ssRNA target. Duplex DNA substrates containing a sequence complementary or noncomplementary to the crRNA were not cleaved (Fig 6A). In contrast, complementary or noncomplementary ssDNA substrates were both cleaved (Fig 6A). These results show that TthCsm cleaves ssDNA but not dsDNA. When we tested a dsDNA substrate containing a 40-nt long region of single-stranded DNA flanked by 24-nt long duplex regions, the central area where ssDNA was exposed was cleaved, whereas minimal cleavage occurred in the flanking regions, which were double-stranded. This indicates that TthCsm can cleave exposed ssDNA in the context of dsDNA, and can act endonucleolytically, since there are no free ssDNA ends in this substrate. A low amount of cleavage occurred in the flanking region, and may be due to the lower T_m_ of the 24 nt duplex regions, compared to the 88-nt duplexes (Fig 6A).

We next investigated the sequence specificity of ssDNA cleavage using short 25-nt ssDNA substrates with different nucleotide compositions (Fig 6B). We compared TthCsm-catalyzed cleavage of oligonucleotides containing all four nucleotides, or oligonucleotides with only three of the nucleotide bases (Fig 6B). Though the pattern of cleavage changed for some of the oligonucleotides, all were cleaved, indicating that the Cas10/Csm1 protein functions as a sequence-independent DNase within TthCsm. Taken together, these results suggest that binding of a complementary RNA transcript to TthCsm activates the Cas10/Csm1 HD domain for nonspecific, endonucleolytic cleavage of ssDNA.

### Coordination of DNA and RNA cleavage by TthCsm

**Fig 7:**
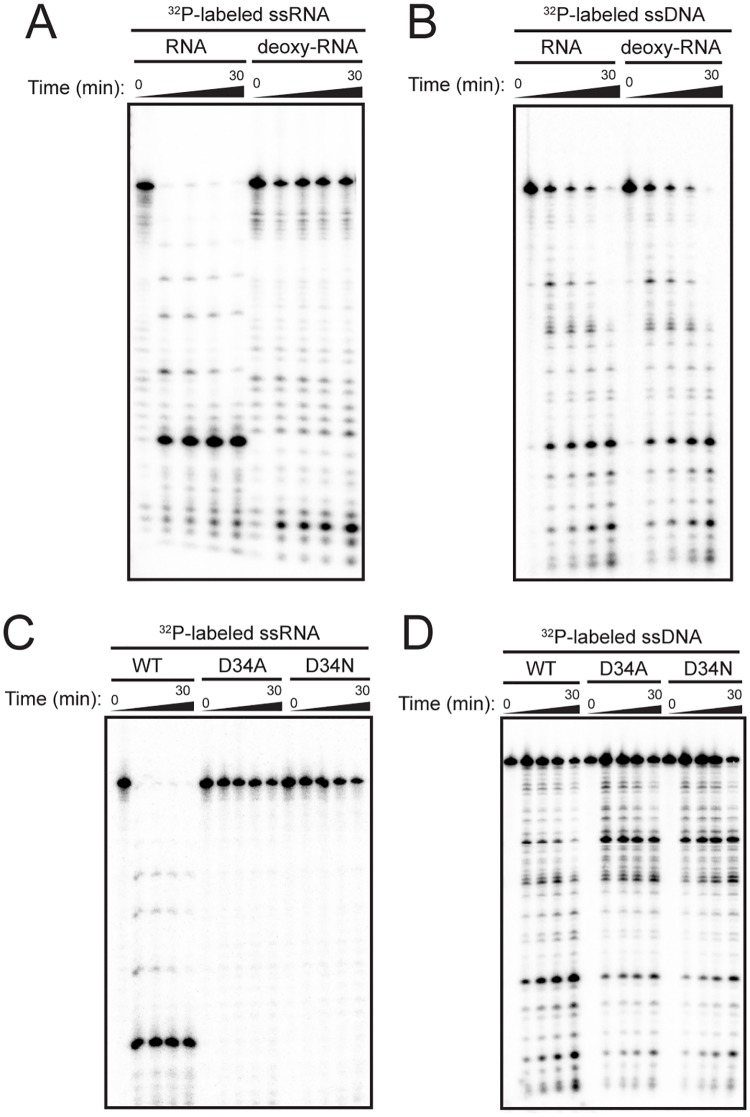
TthCsm-mediated DNA cleavage does not require RNA cleavage. **(A)** TthCsm-mediated cleavage of a complementary ssRNA either with (deoxy-RNA) or without (RNA) deoxynucleotides adjacent to the regularly spaced cleavage sites was tested and analyzed by denaturing PAGE, as in Fig 5C. **(B)** ssDNA cleavage by TthCsm was assayed as in Fig 5B, but with the RNA substrates in (A). **(C)** The wild-type TthCsm (WT) or TthCsm containing a D34A or D34N mutation in the Csm3 subunit (D34A, D34N) were tested for cleavage of a complementary ssRNA target, as in (A). **(D)** WT, D34A, or D34N TthCsm complexes were tested for ssDNA cleavage as in (B).

Next, we asked whether ssDNA cleavage by TthCsm requires RNA cleavage. RNA cleavage by the Type III-B Cmr complex could be blocked by a complementary ssRNA that contains 2’-deoxynucleotides (deoxy-RNA) adjacent to the cleavage sites [11,26]. We tested whether a similar modification of the ssRNA substrate used in this study could also block RNA cleavage by TthCsm. Although binding of a deoxy-RNA substrate complementary to the crRNA in TthCsm was unaffected, cleavage of this deoxy-RNA was not observed (S4 Fig and Fig 7A). When we tested the complementary deoxy-RNA in our DNA cleavage assay, we found that it activated DNA targeting as well as the cleavable, complementary ssRNA (Fig 7B). This indicates that RNA binding but not cleavage is required for DNA cleavage by TthCsm.

We also tested whether TthCsm mutants that are deficient for RNase activity could be activated for DNA cleavage. In Type III-A complexes from *S*. *epidermidis* and *S*. *thermophilus*, a conserved aspartate residue in Csm3 is required for ssRNA cleavage [6,23]. To test if we could generate an RNase-deficient TthCsm complex, we mutated the equivalent D34 residue of *T*. *thermophilus* Csm3 to either alanine or asparagine (D34A or D34N), expressed and purified the mutant complexes, and tested their ssRNA cleavage activity. The mutant complexes could bind but not cleave a complementary ssRNA target (Fig 7C and S4 Fig). When we tested these mutants for ssDNA cleavage in the presence of a target ssRNA, they exhibited a similar activity to the wild-type TthCsm complex, indicating that DNA cleavage does not require cleavage of the ssRNA (Fig 7D). Altogether, this suggests that recognition, but not degradation, of a complementary ssRNA by the complex is necessary for activation of the Cas10/Csm1 subunit for robust DNA cleavage.

## Discussion

Type III CRISPR-Cas systems are among the most common RNA-guided adaptive immune systems in bacteria and archaea, but their mechanisms of target detection and cleavage are not fully understood. *In vivo* and *in vitro* studies indicate that the Type III-A Csm complex from *S*. *epidermidis* mediates targeting of transcriptionally active DNA, but how the target is recognized is not clear, as the complex can degrade both DNA and RNA [6,24]. Type III-A complexes from highly thermophilic microbes, such as *T*. *thermophilus*, present attractive targets for structural and biochemical analysis, owing to the increased stability of proteins that are adapted for function at high temperatures. Until now, however, the TthCsm complex has only been isolated from native hosts, making it difficult to manipulate the protein and crRNA composition of the complex for investigation of the targeting mechanism [21]. Here, we have established the reconstitution of the TthCsm complex in *E*. *coli* with a single crRNA sequence. Using this system, we showed that the TthCsm complex bound and cleaved complemementary ssRNA at a site distinct from that of an ssDNA target. We also found that recognition of a complementary RNA target by TthCsm activates its ssDNA cleavage activity. We identified the HD nuclease domain of the Cas10/Csm1 subunit as the catalytic site for DNA cleavage, and demonstrated that ssDNA was sequence-independent and endonucleolytic. We also generated mutants and used a noncleavable RNA substrate to show that cleavage of the RNA is not necessary for DNA cleavage.

Expression of TthCsm components, Cas6, and a single repeat-spacer-repeat array in *E*. *coli* led to the formation of complexes with different lengths of crRNAs and compositions. Studies of the endogenous TthCsm revealed bound crRNAs of ~35–53 nt [21]. The crRNAs in the reconstituted complex are of similar lengths to those identified in endogenous *T*. *thermophilus* samples, including crRNAs of 42–54 nt representing fully processed guide sequences [21]. We also identified a longer 76 nt-long intermediate, absent from endogenous TthCsm preparations, that may result from inefficient pre-crRNA trimming or incomplete removal of complexes containing partially processed crRNAs during purification [21]. The nuclease that trims crRNAs associated with the reconstituted TthCsm to their mature sizes has not been identified, but the nuclease is likely present in *E*. *coli*. We also found that different lengths of crRNAs corresponded to different numbers of backbone (Csm3) and belly (Csm2) subunits in the complex, consistent with the idea that these subunits assemble along the length of the crRNA. The length of Type I Cascade complexes can also be altered by changing the length of the crRNA, suggesting a conserved mechanism for regulation of complex stoichiometry between Type I and III systems [37–39].

Endogenous TthCsm samples contained some complexes that lacked a Csm5 subunit [21]. We did not observe dissociation of Csm5 in the reconstituted sample, but we found that the Cas10/Csm1 subunit of the complex was dissociated in some of the complexes analyzed by native mass spectrometry, indicating that it may be weakly associated. Whether there is a functional role for an isolated Cas10/Csm1 protein or a TthCsm lacking Cas10/Csm1 in Type III systems is unknown. Like the analogous large subunit, Cas8, of CRISPR Type I-E Cascade complexes, the absence of Cas10/Csm1 did not affect the stoichiometry of the other TthCsm components [40,41]. In the *Pyrococcus furiosus* (*P*. *furiosus)* Type III-B system, the isolated Cas10/Cmr2 subunit is an active DNase, but when it is incorporated into the Cmr complex, it is inactive until the complex is provided with a complementary RNA [15]. The isolated *Thermococcus onnureus* Cas10/Csm1 protein, which also contains an HD domain and palm polymerase domain, is also able to cleave ssDNA [7]. Interaction of a Cas10 protein with the rest of the complex in the absence of a complementary RNA may regulate its DNase activity and prevent nonspecific cleavage of ssDNA in the cell that is exposed, for instance, in DNA replication intermediates. However, at the site of target transcription, dissociation of Cas10/Csm1 from the activated complex may enable it to load onto the ssDNA for more efficient cleavage, perhaps similarly to how the Type I Cas3 nuclease/helicase moves processively along the ssDNA to degrade it [42–44]. Re-association of Cas10/Csm1 with the rest of the complex after the cleaved RNA is dissociated from the complex would once again inhibit its nonspecific ssDNA cleavage activity. Taken together, these results demonstrate that a single Type III CRISPR array from *T*. *thermophilus* can be transcribed, processed into mature crRNAs, and assembled into a TthCsm with only the complex proteins and a Cas6 protein from *T*. *thermophilus*. Efficient production and assembly of Csm targeting complexes *in vivo* may explain in part why Type III-A CRISPR systems have been widely disseminated among microbes.

Analysis of the reconstituted TthCsm sample revealed that nonspecific ssDNA degradation is triggered by complementary RNA, an activity that had not previously been shown for the Type III-A system from this species [21]. We found that binding of an RNA molecule complementary to the crRNA guide sequence within TthCsm is required for sequence-independent DNA cleavage. This is similar to activities reported by Type III-B Cmr complexes from *P*. *furiosus* and *Thermotoga maritima* (*T*. *maritima)* and the Type III-A complex of *S*. *thermophilus*, suggesting that this may be a universal function of Type III effector complexes. Studies of the *S*. *thermophilus* Csm and the *T*. *maritima* Cmr also indicate that cleavage of the RNA inactivates the DNA targeting activity of the complex, which may prevent further cleavage of exposed ssDNA in the cell [10,11]. The *S*. *thermophilus* Csm, however, was still able to degrade a circular ssDNA slowly, but completely, in the absence of RNA, while the TthCsm catalyzed virtually no cleavage when complementary RNA was not present (Fig 4B and S2 Fig) [10]. Whether this basal cleavage activity of some Type III-A complexes is harmful to cells or whether additional regulatory factors might be involved is unknown.

Complementarity of the 5′ tag of the crRNA to the target DNA has been proposed to allow Type III CRISPR-Cas systems to distinguish self from non-self DNA, as the 5′ tag is derived from the CRISPR repeat sequence [36,45]. A recent study of a mesophilic *S*. *thermophilus* Csm complex indicated that the recognition may instead be read in the ssRNA [10]. Here, we found that TthCsm-mediated DNA cleavage requires a lack of complementarity between the 3′ flanking sequence of the target RNA and the 5′ tag of the crRNA. This could prevent cleavage of host DNA by Type III CRISPR-Cas complexes if transcription occurs across in an antisense direction across the CRISPR loci [10]. Our findings suggest that binding of the target RNA to TthCsm positions the 3′ flanking region over the surface of the Cas10/Csm1 protein in such a way that results in its activation. However, since an 8-nt oligo did not activate the TthCsm in *trans*, this interaction is likely weak and requires a covalent connection with a bound RNA target sequence. Structural studies will be needed to identify the conformational changes and interactions that lead to activation of Cas10/Csm1. This RNA-triggered nonspecific DNA cleavage activity is reminiscent of the mechanism of the Type VI CRISPR effector protein, C2c2, which is activated for nonspecific RNA cleavage upon binding to its cognate RNA target sequence [17,20]. Thus, further studies of the targeting mechanism in detail may reveal new parallels between different types of CRISPR-Cas systems.

By reconstituting mutants of the TthCsm complex using our *E*. *coli* system, we also uncovered the active site for DNA cleavage activity. Similar to studies of the reconstituted *P*. *furiosus* Cmr, *T*. *maritima* Cmr, and the *S*. *thermophilus* Csm complexes, we find that TthCsm requires the Cas10/Csm1 HD domain’s active site for DNA cleavage, but not the palm polymerase catalytic residues [10,11,15]. However, *in vivo* and *in vitro* studies of the Csm complex from *S*. *epidermidis* suggest that the conserved GGDD motif of the palm polymerase domain is required for DNA targeting [6,25]. The HD motif was not tested in the *in vitro* assay, but it is possible that the perturbation of the palm polymerase domain may destabilize the stability of the protein fold in some Cas10 proteins. Perhaps owing to the thermostable nature of TthCsm, we did not observe significant effects of the GGDD mutant on complex stability or RNA binding and cleavage. Thus, the HD domain is the most likely catalytic site for DNA cleavage in Type III complexes.

**Fig 8:**
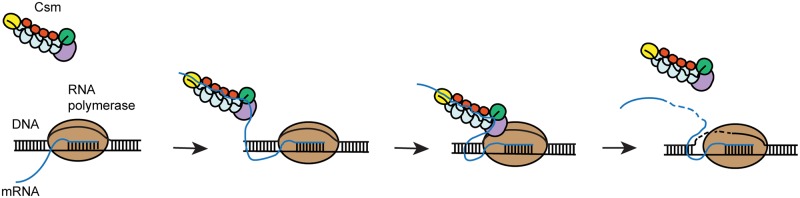
A conserved mechanism for co-transcriptional DNA and RNA targeting by Type III-A CRISPR-Cas effector complexes. During transcription, RNA polymerase transiently unwinds double-stranded DNA as it synthesizes a messenger RNA transcript (mRNA) that is complementary to the template strand. Transcription across a region of the genome that is complementary to the crRNA leads to the production of an mRNA containing a region complementary to the crRNA in the Csm complex. The Csm complex would recognize and bind the mRNA through base-pairing interactions with its crRNA, leading to activation of the sequence-independent DNA endonuclease activity of the HD domain in Csm1. This would lead to Csm-mediated cleavage of transiently unwound ssDNA, while Csm is tethered via the RNA. Self-targeting is avoided by preventing cleavage when the 3′ flanking region of the RNA is complementary to the 5′ crRNA tag. Following DNA and RNA cleavage, the Csm complex dissociates from its targets.

We also identified the nature of TthCsm’s preferred DNA substrate. TthCsm targeted ssDNA but not dsDNA, and a mispaired region within a dsDNA substrate was also cleaved, indicating that the complex possesses endonucleolytic activity. DNA cleavage was specific for ssDNA but not dsDNA, and a mis-paired region within a dsDNA substrate was also cleaved, indicating that the HD domain possesses endonucleolytic activity. Interestingly, dsDNA cleavage activity was reported in a *P*. *furiosus* Cmr, but it is possible this may have been due to transient melting of the duplex DNA at the high temperatures used for the assay (70°C) [15]. Taken together, this study and others support a mechanism in which transcription-dependent ssDNA cleavage by Type III systems occurs only as RNA polymerase unwinds dsDNA during transcription and generates an RNA transcript containing a crRNA-complementary target sequence (Fig 8). The requirement for complementary RNA binding to activate DNA cleavage would prevent the complex from cleaving ssDNA at off-target sites in the cell [10,11]. A portion of the unwound nontemplate strand is solvent-exposed in structures of bacterial transcription complexes and is accessible to cleavage by nucleases, suggesting it may be possible for the Cas10 subunit in TthCsm to cleave directly at the transcription bubble [46,47]. However, direct evidence for an interaction with an assembled transcription bubble containing the RNA polymerase has not yet been shown. Taken together, these results lead to a conserved mechanism used by Type III-A and III-B CRISPR systems in which sequence-specific recognition of ssRNA binding triggers localized but non-sequence-specific ssDNA cleavage.

This reconstitution of a highly thermostable Type III-A Csm complex in *E*. *coli* with a single crRNA sequence is a valuable tool for future structural investigations of DNA and RNA targeting by Type III CRISPR-Cas systems. The fact that a defined crRNA sequence is associated with the complex makes it easier to generate target-bound complexes for structural studies. In addition, the thermostability of the TthCsm makes it an attractive model system for X-ray crystallography and/or cryo-EM studies, and the complex has already been shown to be amenable to negative-stain EM analysis [21]. In contrast, the *S*. *thermophilus* Csm, which has also been reconstituted *in vivo*, was only shown to be active at 37°C, and its natural host grows within a range of 35–42°C [10,23,48]. Our system also has an advantage over an endogenous system in that we can perform routine mutagenesis of the TthCsm complex. We also found that the catalytic requirements for DNA and RNA targeting by this complex are similar to those of Csm and Cmr complexes from other species, suggesting that studies of target recognition and cleavage using this system would have a broad impact. Determination of the mechanism of transcription-dependent DNA and RNA targeting by Type III systems could lead to the discovery of unexpected similarities with other CRISPR systems that target RNA, like the Type VI C2c2 effector, and deepen our understanding of the evolution of CRISPR-Cas systems in prokaryotes.

## Supporting Information

S1 Fig Native mass spectrometry of reconstituted TthCsm complexes. Native nanoelectrospray ionization mass spectrometry (nanoESI-MS) was performed on the reconstituted TthCsm. Measured molecular masses of the complexes are listed in the top right corner. Ions of the different complexes are labeled using different colors. Cartoons of the complex stoichiometries for each mass detected are shown on the right.
(TIF)

S2 Fig The TthCsm complex mediates minimal ssDNA cleavage in the presence of a complementary ssRNA target and MgCl_2_. TthCsm-mediated ssDNA cleavage was monitored in the presence of complementary (C) and noncomplementary (NC) ssRNA, as in Fig 4, but with 5 mM MgCl_2_ instead of 5 mM MnCl_2_.
(TIF)

S3 Fig Loss of ssDNA cleavage activity in the TthCsm complex by truncation of the 3’ flanking region of the activator RNA cannot be reversed by a short noncomplementary ssRNA oligo added in *trans*. The TthCsm complex (200 nM) was incubated with 200 nM complementary ssRNA with a truncated 3′ flanking region (Δ3′) and 5 nM 5′-^32^P-radiolabeled ssDNA in the presence of MnCl_2_, and cleavage products were analyzed by denaturing PAGE, as in Fig 4. Where indicated, an 8-nt oligonucleotide that was not complementary to the 5’ tag of the crRNA was also included at a 10-fold or 100-fold molar excess over the complementary ssRNA.
(TIF)

S4 Fig Related to Fig 7. EMSA experiments were performed to test binding of mutant TthCsm complexes (D34A or D34N mutation in Csm3 subunit) with complementary RNA (D34A, D34N), and wild-type TthCsm with deoxy-RNA substrate. RNA cleavage was inhibited by the omission of metal ions, and inclusion of 1 mM EDTA.
(TIF)

S1 Table DNA and RNA oligonucleotides used and their sequences. (TIF)

S2 Table *Thermus thermophilus* Csm proteins and *E*. *coli* GroEL detected in the reconstituted TthCsm sample by mass spectrometry. (TIF)
