# Correction: RNA and DNA Targeting by a Reconstituted *Thermus thermophilus* Type III-A CRISPR-Cas System

**DOI:** 10.1371/journal.pone.0175612

**Published:** 2017-04-06

**Authors:** Tina Y. Liu, Anthony T. Iavarone, Jennifer A. Doudna

The following information is missing from the Funding section: A mass spectrometer used in this study was purchased using NIH Shared Instrumentation Grant 1S10OD020062-01.
