# Supplementary figures and images for: RNA and DNA Targeting by a Reconstituted Thermus thermophilus Type III-A CRISPR-Cas System
Source: PLoS One. 2017 Jan 23;12(1):e0170552. doi: 10.1371/journal.pone.0170552 (PMC5256923; doi:10.1371/journal.pone.0170552)

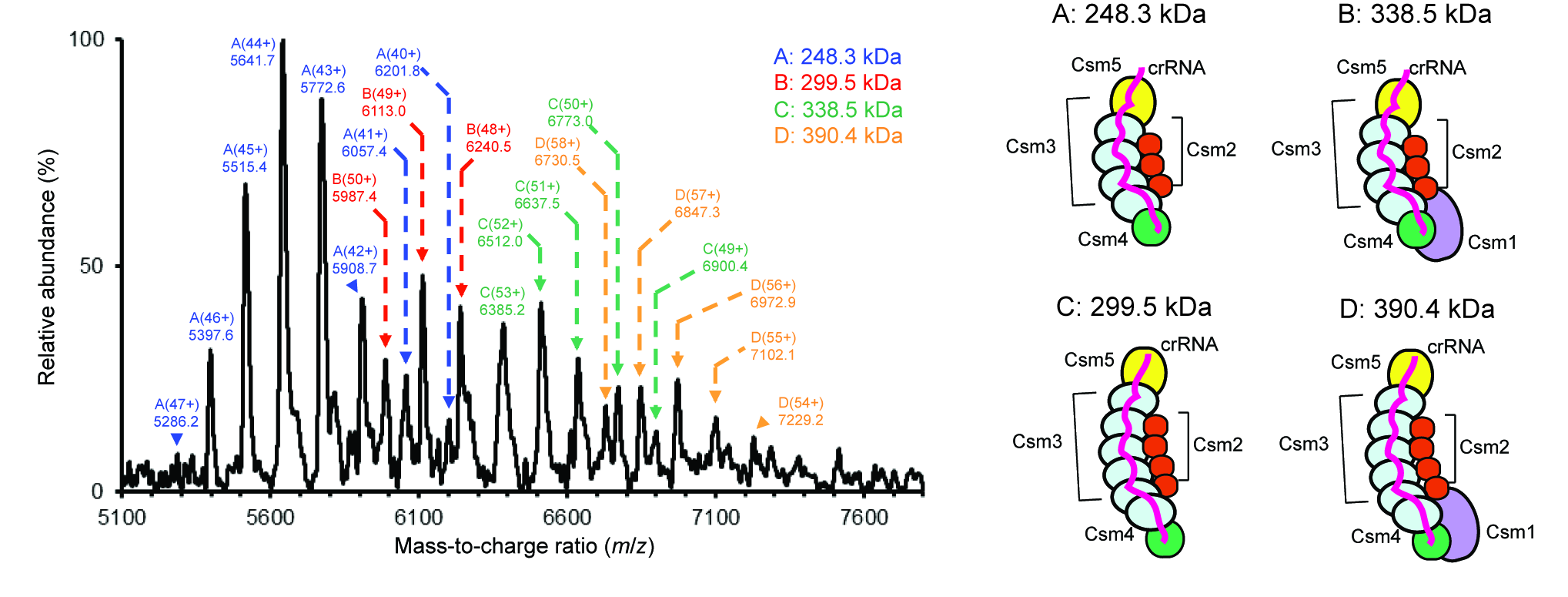

Supplement: S1 Fig — Native nanoelectrospray ionization mass spectrometry (nanoESI-MS) was performed on the reconstituted TthCsm. Measured molecular masses of the complexes are listed in the top right corner. Ions of the different complexes are labeled using different colors. Cartoons of the complex stoichiometries for each mass detected are shown on the right. (TIF) [file pone.0170552.s001.tif]

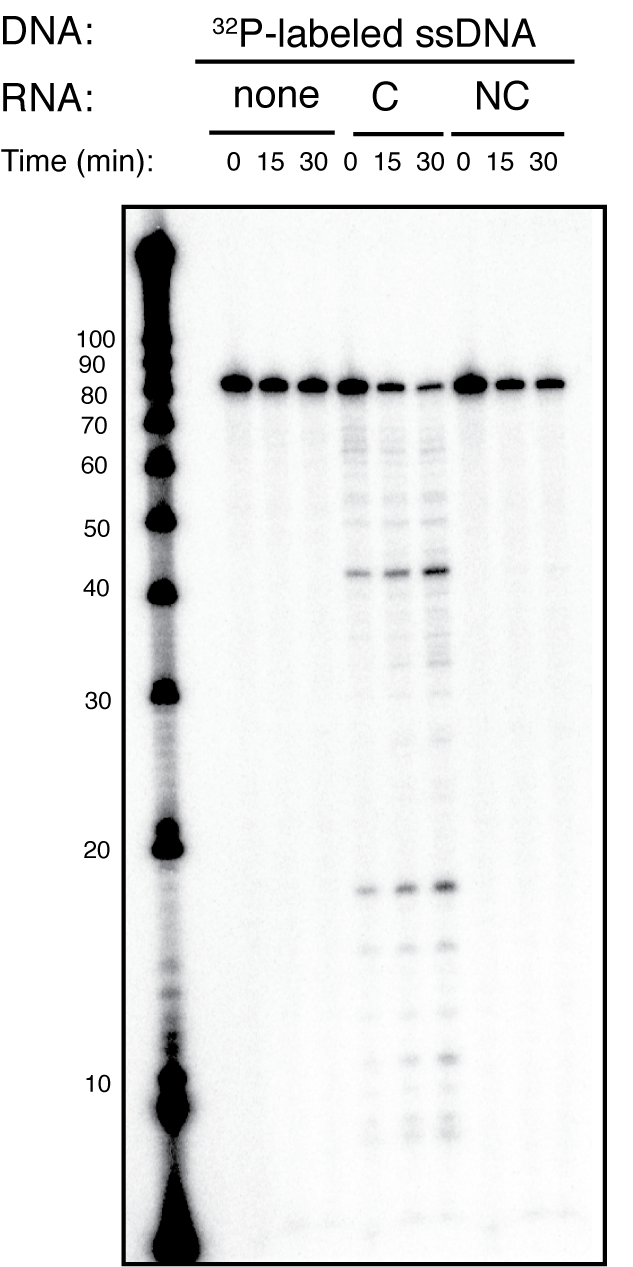

Supplement: S2 Fig — TthCsm-mediated ssDNA cleavage was monitored in the presence of complementary (C) and noncomplementary (NC) ssRNA, as in Fig 4, but with 5 mM MgCl2 instead of 5 mM MnCl2. (TIF) [file pone.0170552.s002.tif]

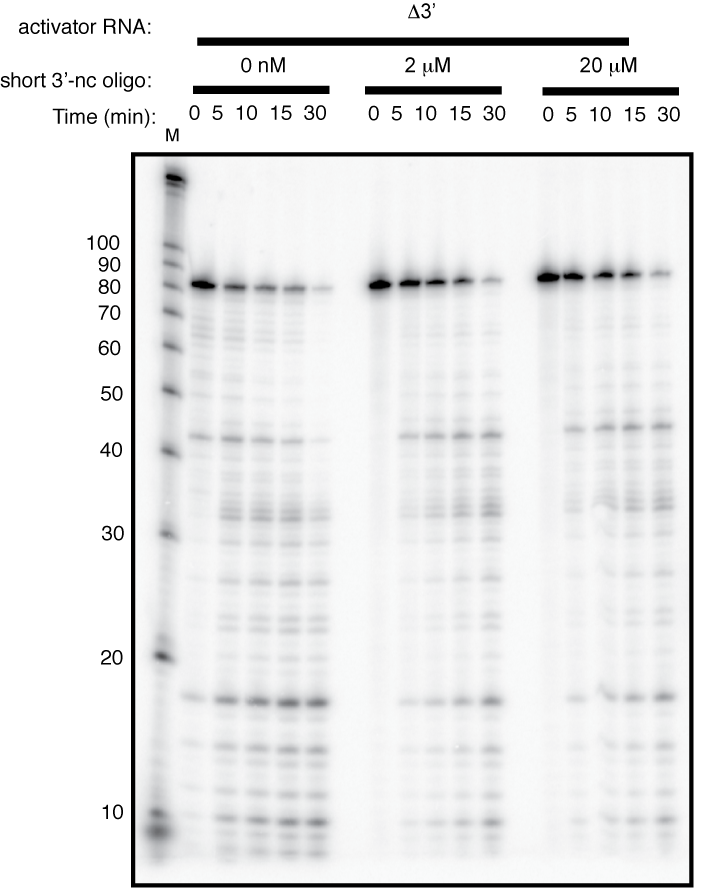

Supplement: S3 Fig — The TthCsm complex (200 nM) was incubated with 200 nM complementary ssRNA with a truncated 3′ flanking region (Δ3′) and 5 nM 5′-32P-radiolabeled ssDNA in the presence of MnCl2, and cleavage products were analyzed by denaturing PAGE, as in Fig 4. Where indicated, an 8-nt oligonucleotide that was not complementary to the 5’ tag of the crRNA was also included at a 10-fold or 100-fold molar excess over the complementary ssRNA. (TIF) [file pone.0170552.s003.tif]

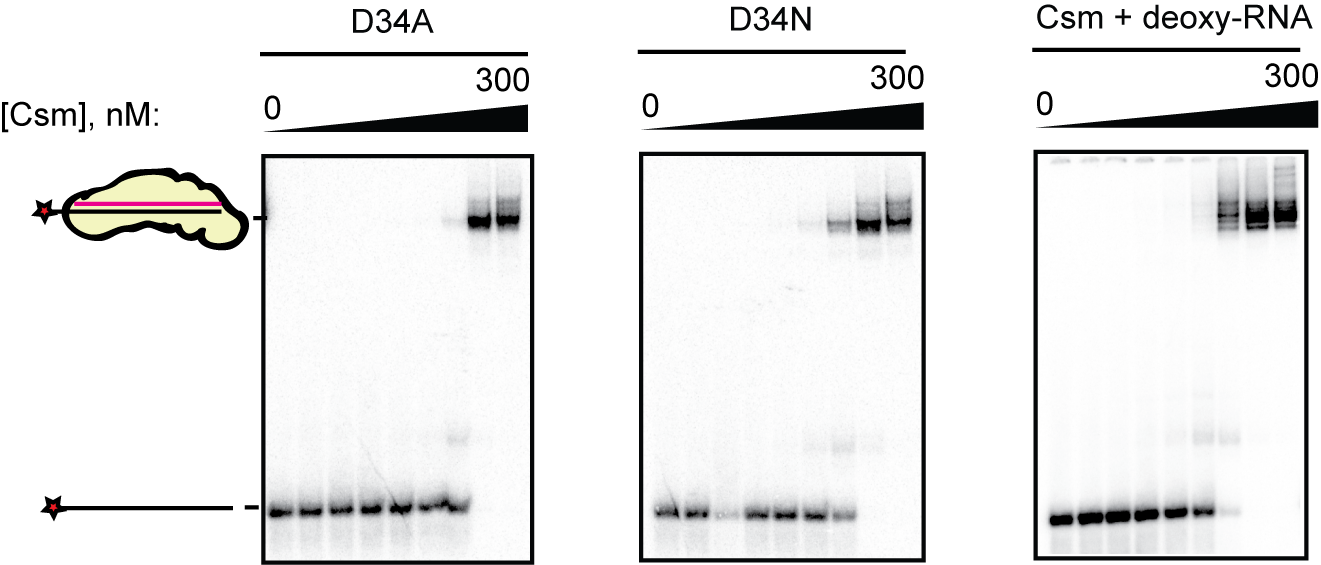

Supplement: S4 Fig — EMSA experiments were performed to test binding of mutant TthCsm complexes (D34A or D34N mutation in Csm3 subunit) with complementary RNA (D34A, D34N), and wild-type TthCsm with deoxy-RNA substrate. RNA cleavage was inhibited by the omission of metal ions, and inclusion of 1 mM EDTA. (TIF) [file pone.0170552.s004.tif]

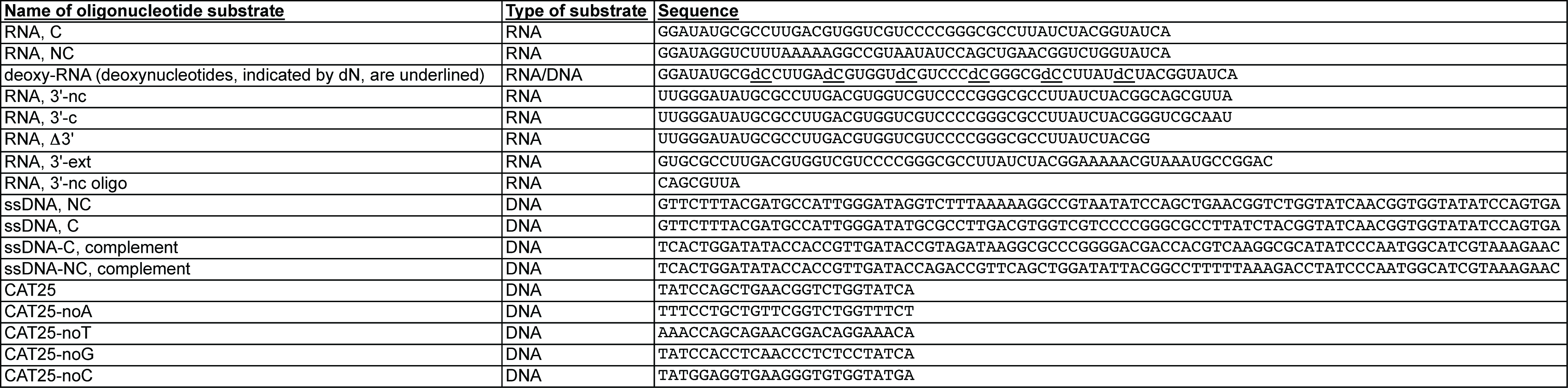

Supplement: S1 Table — (TIF) [file pone.0170552.s005.tif]

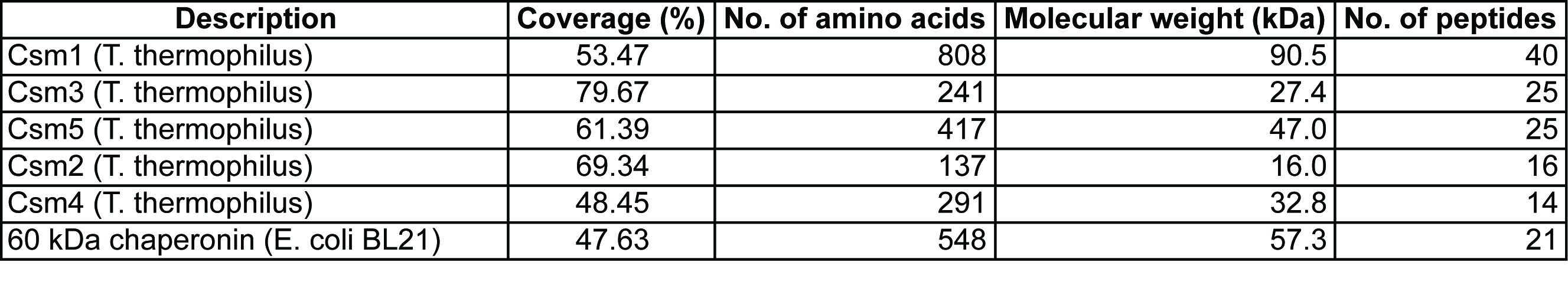

Supplement: S2 Table — (TIF) [file pone.0170552.s006.tif]
